# Public Opinions about Overdiagnosis: A National Community Survey

**DOI:** 10.1371/journal.pone.0125165

**Published:** 2015-05-20

**Authors:** Ray Moynihan, Brooke Nickel, Jolyn Hersch, Elaine Beller, Jenny Doust, Shane Compton, Alexandra Barratt, Lisa Bero, Kirsten McCaffery

**Affiliations:** 1 Centre for Research in Evidence-Based Practice, Bond University, Robina, Queensland, Australia; 2 School of Public Health, University of Sydney, Sydney, New South Wales, Australia; 3 The Social Research Centre, Melbourne, Victoria, Australia; 4 Charles Perkins Centre, University of Sydney, Sydney, New South Wales, Australia; Department of Transplantation and Renal Medicine, AUSTRALIA

## Abstract

**Background:**

Despite evidence about the "modern epidemic" of overdiagnosis, and expanding disease definitions that medicalize more people, data are lacking on public views about these issues. Our objective was to measure public perceptions about overdiagnosis and views about financial ties of panels setting disease definitions.

**Methods:**

We conducted a 15 minute Computer Assisted Telephone Interview with a randomly selected community sample of 500 Australians in January 2014. We iteratively developed and piloted a questionnaire, with a convenience sample (n=20), then with participants recruited by a research company (n=20). Questions included whether respondents had been informed about overdiagnosis; opinions on informing people; and views about financial ties among panels writing disease definitions.

**Findings:**

Our sample was generally representative, but included a higher proportion of females and seniors, typical of similar surveys. American Association for Public Opinion Research response rate was 20% and cooperation rate was 44%. Only 10% (95% CI 8%–13%) of people reported ever being told about overdiagnosis by a doctor. 18% (95% CI 11%–28%) of men who reported having prostate cancer screening, and 10% (95% CI 6%–15%) of women who reported having mammography said they were told about overdiagnosis. 93% (95% CI 90%–95%) agreed along with screening benefits, people should be informed about overdiagnosis. On panels setting disease definitions, 78% (95% CI 74%–82%) felt ties to pharmaceutical companies inappropriate, and 91% (95% CI 82%–100%) believed panels should have a minority or no members with ties. Limitations included questionnaire novelty and complexity.

**Conclusions:**

A small minority of Australians surveyed, including those reporting being screened for prostate or breast cancer, reported being informed of overdiagnosis; most believed people should be informed; and a majority felt it inappropriate that doctors with ties to pharmaceutical companies write disease definitions. Results suggest strategies to better inform people about overdiagnosis, and review disease definition processes, have significant public sympathy.

## Introduction

The “modern epidemic” of overdiagnosis is now recognised as an important risk to health [1, 2], with evidence-based efforts underway to combat it [3]. Overdiagnosis occurs when someone is diagnosed with a disease that would not have harmed them [2], often as a result of undergoing screening, and evidence is emerging that many people are overdiagnosed and labelled unnecessarily across a range of conditions [4]. An inquiry in the United Kingdom estimated 19% of the breast cancers detected during mammography screening may be overdiagnosed [5], and the United States Preventive Services Task Force recently noted there is “convincing evidence that PSA-based screening leads to substantial overdiagnosis of prostate tumors”, with estimates ranging from 17% to 50% [6].

There are on-going scientific discussions about the best methods for measuring overdiagnosis [7], as well as strong arguments that some degree of overdiagnosis is an inevitable risk of screening programmes [8], and that attempts to prevent it should not come at the cost of increasing under-diagnosis. Notwithstanding these debates, there is now official recognition of the need for greater awareness of the problem. As a working group convened under the auspices of the National Cancer Institute has observed, “Physicians, patients, and the general public must recognize that overdiagnosis is common and occurs more frequently with cancer screening” [9].

Screening programs are one of many causes of overdiagnosis including technological changes enabling detection of ever-smaller abnormalities, commercial interests seeking wider markets, the medicalization of risk and cultural enthusiasm for early detection [2]. Overdiagnosis can also be seen as one aspect of much broader processes of biomedicalization [10]. “In the biomedicalization era” wrote sociologists Clarke and colleagues “what is perhaps most radical is the biomedicalization of health itself”, an era when “it is no longer necessary to manifest symptoms to be considered ill or ‘at risk’”[10]. Armstrong has also described the inexorable rise of “surveillance medicine”, which reconstructs the nature of disease to become “less the illness per se but rather the semi-pathological pre-illness at-risk state” [11]. More recently, in a series in *The BMJ*, researchers are investigating how expanding disease definitions which label more people with milder symptoms or at lower risks are increasing the potential for overdiagnosis, with examples including thyroid cancer [12], gestational diabetes [13], and pulmonary embolism [14].

A 2013 study of the guideline panels which recently changed definitions of 14 common conditions found a majority widened those definitions—including creating pre-hypertension, expanding the diagnosis of myocardial infarction, and lowering diagnostic thresholds for Attention Deficit Hyperactivity Disorder [15]. In addition panel publications did not generally report potential harms of these changes, including risks of overdiagnosis, and among panels which made disclosures, 75% of members had multiple ties to pharmaceutical companies, including those benefiting directly from any increase in populations classified as patients. This finding of extensive conflicts of interest among medical professionals who define human disease is in stark contrast to recommendations from the Institute of Medicine that guideline panels should wherever possible exclude members with conflicts [16].

While overdiagnosis and expanding disease definitions are recognised as important and related problems, data on public awareness and views about them are extremely limited. In 2004, Schwartz and colleagues found widespread enthusiasm for cancer screening, largely unmodified by awareness of potential harms [17]. More recently Hersch and colleagues published focus group data on Australian women’s views on overdiagnosis of breast cancer, finding high enthusiasm for screening and minimal awareness of overdiagnosis, but also a demand for information about the topic [18]. In 2013 an on-line survey of 317 people invited to cancer screening found under 10% were informed by their doctor about the risk of overdiagnosis and overtreatment, and 80% expressed a desire to be informed about these risks [19]. To our knowledge, no previous survey has asked the general community about perceptions and views on overdiagnosis. And while there is data on public views about different aspects of industry-health professional relationships [20, 21], no study has sought community views specifically about ties of panels which change disease definitions.

We aimed to measure the general community’s awareness and perceptions about overdiagnosis and views about financial ties of panels which set disease definitions and diagnostic thresholds. Notwithstanding important limitations outlined below, we believe our results will help inform attempts to better communicate about overdiagnosis.

## Methods

We conducted a national Computer Assisted Telephone Interview (CATI) survey with 500 members of the Australian community aged 18 years and older, using a randomly selected dual frame sample—including land-line and mobile phones—during January and February 2014. The survey questionnaire included items about awareness of overdiagnosis, experience of being informed about overdiagnosis during screening, enthusiasm for genetic screening—a possible pathway to overdiagnosis [22]—and attitudes to financial ties of expert panel members who change disease definitions. It also collected demographic information on age, gender, employment, education, and cancer history. Questions were iteratively developed jointly by all authors, based on published and unpublished findings including from focus groups on views about overdiagnosis with 50 women of diverse age and educational background, [18] a qualitative study on patient attitudes [23], and the 2004 survey of attitudes towards screening [17].

Draft items were piloted initially by three authors (RM, BN, JH) with a convenience sample of 20 adults. Then 20 pilot telephone interviews were conducted by an experienced social research company, the Social Research Centre, which subsequently conducted 500 interviews.

The survey sample size of 500 was chosen as appropriately powered so that the confidence interval around the proportion responding affirmatively would be approximately 4% either side of the observed proportion for the expected responses to key questions on awareness of overdiagnosis (expected response around 20%), enthusiasm for screening (expected response around 80%), and belief people should be made aware of risks (expected response around 80%).

A dual frame random digit dialling sample design was employed with a 50:50 split between landline and mobile samples. After calling the randomly selected telephone numbers, interviewers asked to speak with the person in the household aged 18 years or over who had the last birthday (landlines) or confirmed if the person answering was over 18 years (mobiles). A slightly modified approach was adopted after approximately 400 interviews, in order to target more difficult to reach demographic groups, notably males and young adults. Rather than asking for the person with the last birthday, the modified screening approach requested to speak with the youngest adult male. Once a potential interview was established, interviewers provided information about the research purpose and process, and obtained informed consent. (see Ethics Statement below) Answer options included yes/no answers, and Likert type scales to offer more options for intensity of response.

The survey took approximately 15 minutes to complete. Key questionnaire questions and the brief explanation of overdiagnosis offered to participants after the question on unprompted awareness are listed in Table 1. Other questions are available online (S1 File). An open-ended item and a separate section on concern and treatment preferences relating to ductal carcinoma in situ terminology are being separately analysed and reported elsewhere. At the questionnaire conclusion, participants were asked if they would like to participate in a follow-up qualitative interview about similar topics, and if so, provide name and contact details.

**Table 1:** Survey Questions.

Survey Question	Response Format
**On awareness and opinions about overdiagnosis**	
Have you seen or heard the term ‘over-diagnosis’ before today?	Yes
	No
	Don’t know
A generally accepted view is that over-diagnosis happens when people are diagnosed with a disease that would never have harmed them. This could be due to the condition being so slow developing or them displaying only very minor symptoms. Given this explanation, have you seen or heard the term or concept of ‘over-diagnosis’?	Yes
	No
	Don’t Know
Has a doctor ever told you that healthy people can be over-diagnosed as a result of being screened or tested for a disease?	Yes
	No
	Don’t Know
[For those who reported being screened for prostate or breast cancer]: Were you told about the risk of over-diagnosis?	Yes
	No
	Don’t Know
Do you think routine screening tests for healthy people are almost always a good idea?	Yes
	No
	Don’t Know
When healthy people are considering having a screening test—along with being told about the potential benefits of the screening test—do you agree or disagree that they should be informed about the potential risk of over-diagnosis?	7 point Likert scale:
	Completely Agree
	to
	Completely Disagree
**On enthusiasm for genetic screening**	
Imagine that there was a genetic screening test which could analyse your genes and identify all the diseases you may ever get, for which some had effective treatments and some did not. Would you be likely or unlikely to have that screening test?	7 point Likert scale:
	Completely Likely
	To
	Completely Unlikely
Imagine now that the results of the genetic screening test were often uncertain, and the predictions could be wrong. Would you be likely or unlikely then to have that screening test?	7 point Likert scale:
	Completely Likely
	To
	Completely Unlikely
**On expert disease panel ties to pharmaceutical companies**	
From time to time, doctors who specialise in a particular disease will come together to discuss the characteristics of that disease, to decide who should be diagnosed with it and who requires treatment for it. These are called panels and currently some doctors on these panels have financial ties with pharmaceutical companies who market drugs for that disease and some do not. Is it appropriate or inappropriate for doctors who have financial ties with pharmaceutical companies to be members of these panels?	7 point Likert scale:
	Completely Appropriate
	to
	Completely Inappropriate
Ideally, what proportion of the panel should be made up of doctors with financial ties to pharmaceutical companies who market drugs for that disease?	None
	A minority
	A majority

There is debate in the survey literature about different ways to calculate outcome rates, with a key question being to what extent households that could not be contacted or screened are included in the denominator. To assess our sampling strategy we calculated the response rate and cooperation rate as per recommendations and formulae from the American Association for Public Opinion Research [24]. The AAPOR response rate includes in its denominator estimations of the proportion of cases of unknown eligibility which is actually eligible, and calculations involve all households including those where no contact at all was made. The AAPOR cooperation rate excludes un-contacted households, and calculates the proportion of those contacted who cooperated.

No weighting was applied to primary results. For adjusted results, a two-stage weighting process was used where-by a pre-weight to adjust for the overlapping sample was calculated for people with and without a mobile phone. People have varying chances of selection in a dual-frame sample and those with a landline and mobile phone have multiple chances of selection. After these pre-weights were calculated, post-stratification weights were created using rim weighting to adjust weighted proportions to comply with population proportions from four benchmarks obtained from the Australian Bureau of Statistics for gender, age, location and education [25,26]. All results were analyzed descriptively using IBM SPSS Statistics 22, using proportions and confidence intervals. Chi-square tests of association were used to determine the strength of association between demographic variables and four key questions. Variables significant at the 5% level in chi-square analyses were fitted in multivariable models.

### Ethics Statement

Ethics approval was granted by the Bond University Human Research Ethics Committee, BUHREC, whose comments helped refine questionnaire text. (Approval #RO1765) Participants were assured responses would be anonymous and not recorded, and in order to maximise informed consent, a Participant Information Sheet was developed and made available to be read on request, and posted on accessible websites. The information sheet and the process for seeking informed consent were explicitly approved by BUHREC. Interviewers underwent a tailored training session in preparation for the survey, covering topics including sensitive subject matter training and strategies for handling distressed respondents.

## Results

**Fig 1:**
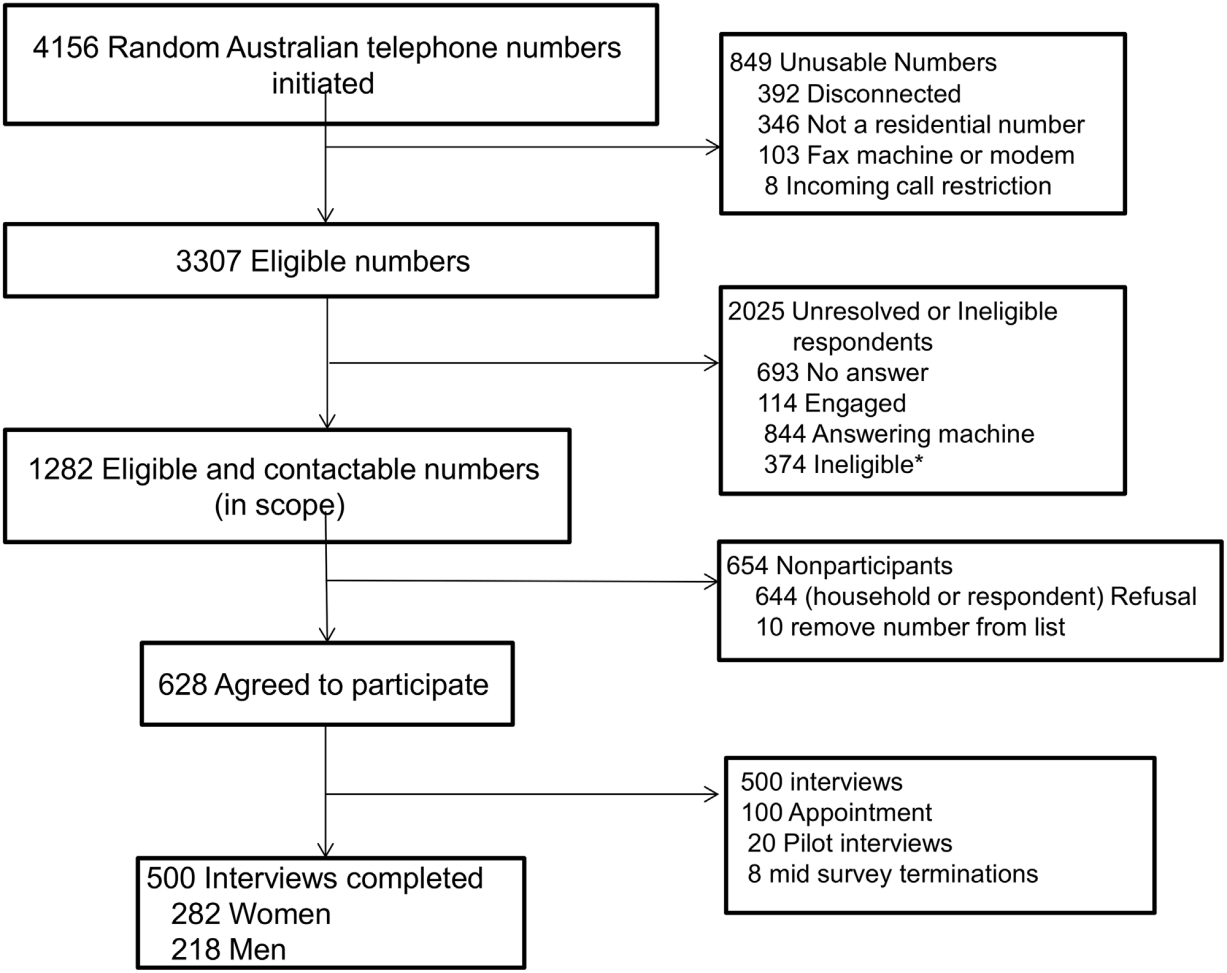
Participant recruitment for Computer Assisted Telephone Interview survey of 500 Australians. *Ineligible participants included: persons under age 18 years; those with a medical condition rendering them physically unable to complete the interview; people with language difficulties; respondent away for duration of fieldwork; people claiming to have done survey or named person not known.

The random sample selection process commenced with 4,268 numbers available, from which 4,156 landline and mobile calls were initiated, and 3,307 eligible numbers identified. Contact was made with 1,282 numbers from which 500 completed interviews were achieved, 251 from the landline sample and 249 from the mobile phone sample, in addition to the 20 pilot interviews, and 8 mid-survey terminated interviews (Fig 1). The response rate was 20.4% (AAPOR, RR3) and the cooperation rate 43.8% (AAPOR, COOP3).

The sample was generally representative, but included a higher proportion of women and older adults than the general Australian community, as is typical with telephone based health surveys, and slightly higher levels of education. (Table 2) All proportions reported here are unadjusted, and both adjusted and unadjusted results are available in Table 3, demonstrating generally minimal impact of adjustment.

**Table 2:** Characteristics of survey respondents.

Characteristic	No. of Survey Respondents n = 500 (%)
Age, y	
18–29	76 (15.2) * *(21.4)*
30–49	139 (27.8) * *(36.5)*
50–69	209 (41.8) * *(29.5)*
≥70	76 (15.2) * *(12.7)*
Sex	
Men	218 (43.6) * *(49.4)*
Women	282 (56.4) * *(50.6)*
Education **	
<High school	74 (14.8) * *(26.9)*
High school graduate	169 (33.8) * *(38.7)*
Bachelor degree/advanced diploma	168 (33.6) * *(26.5)*
>Bachelor degree	89 (17.8) * *(7.7)*
Employment	
Employed	298 (59.6)
Unemployed	20 (4)
Not working***	182 (36.4)
Cancer diagnosis	
Yes	70 (14.0)
No	430 (86.0)

*Australian population data from the Australian Bureau of Statistics 2011 Census;

** High school normally completed at age 17;

*** Not in labour force (e.g. student, retired)

**Table 3:** Main results of national community survey on overdiagnosis.

On overdiagnosis	Unadjusted			Adjusted*		
	Yes/Agree	No/Disagree	Don’t know/ neither/refused	Yes/Agree	No/Disagree	Don’t know/ neither/refused
	n(%)(95%CI)	n(%)(95%CI)	n(%)(95%CI)	%(95%CI)	%(95%CI)	%(95% CI)
Seen or heard term ‘overdiagnosis’ before?	313(62.6)(58.2–66.8)	181(36.2)(32.0–40.6)	6(1.2)(0.5–2.7)	57.4(52.9–61.8)	41.1(36.7–45.4)	1.5(0.8–3.3)
Doctor ever told you about overdiagnosis?	52(10.4)(7.9–13.5)	443(88.6)(85.4–91.2)	5(1.0)(0.4–2.5)	9.7(7.2–12.6)	89.5(86.3–91.9)	0.8(0.3–2.2)
If screened for prostate cancer, told of overdiagnosis?	16(18.2)(11.1–28.1)	71(80.7)(70.6–88.0)	1(1.1)(0.1–7.1)	15.8(9.2–26.3)	83.1(72.4–89.9)	1.1(0.1–7.6)
If screened for breast cancer, told of overdiagnosis?	18(9.7)(6.0–15.1)	162(87.1)(81.2–91.4)	6(3.2)(1.3–7.2)	10.8(6.5–17.3)	86.4(79.5–91.3)	2.9(0.9–7.3)
Think routine screening almost always good idea?	382(76.4)(72.4–80.0)	85(17.0)(13.9–20.7)	33(6.6)(4.7–9.2)	79.0(75.1–82.4)	15.1(12.1–18.5)	6.0(4–8.3)
Should people be informed about risk of overdiagnosis?	465(93.0)(90.3–95)	18(3.6)(2.2–5.7)	17(3.4)(2.1–5.5)	93.4(90.8–95.4)	3.5(2.4–6)	3.0(1.9–5.3)
**On genetic screening**	**Likely**	**Unlikely**	**Don’t know/ neither**	**Likely**	**Unlikely**	**Don’t know/neither**
Likely or unlikely to have genetic screening test?	243(48.6)(44.2–53.1)	226(45.2)(40.8–49.7)	31(6.2)(4.3–8.8)	51.0(46.5–55.5)	42.5(38.2–47.1)	6.5(4.7–9.2)
If results uncertain, likely or unlikely to have test?	142(28.4)(24.5–32.6)	335(67.0)(62.7–71.1)	23(4.6)(3–6.9)	31.0(27.2–35.5)	64.0(59.6–68.2)	4.8(3.2–7.2)
**On financial ties of disease-defining panels**	**Appropriate**	**Inappropriate**	**Don’t know/ neither/refused**	**Appropriate**	**Inappropriate**	**Don’t know/neither/refused**
Appropriate or inappropriate for doctors with ties to pharmaceutical companies to be panel members?	71(14.2)(11.3–17.6)	391(78.2)(74.3–81.7)	38(7.6)(5.5–10.4)	16.2(13–19.6)	75.6(71.6–79.3)	8.1(5.8–10.8)

*Adjustment involved two-step, rim weighting as described in Methods; due to rounding some rows do not add to 100%; calculation of Confidence Intervals includes continuity correction

**Fig 2:**
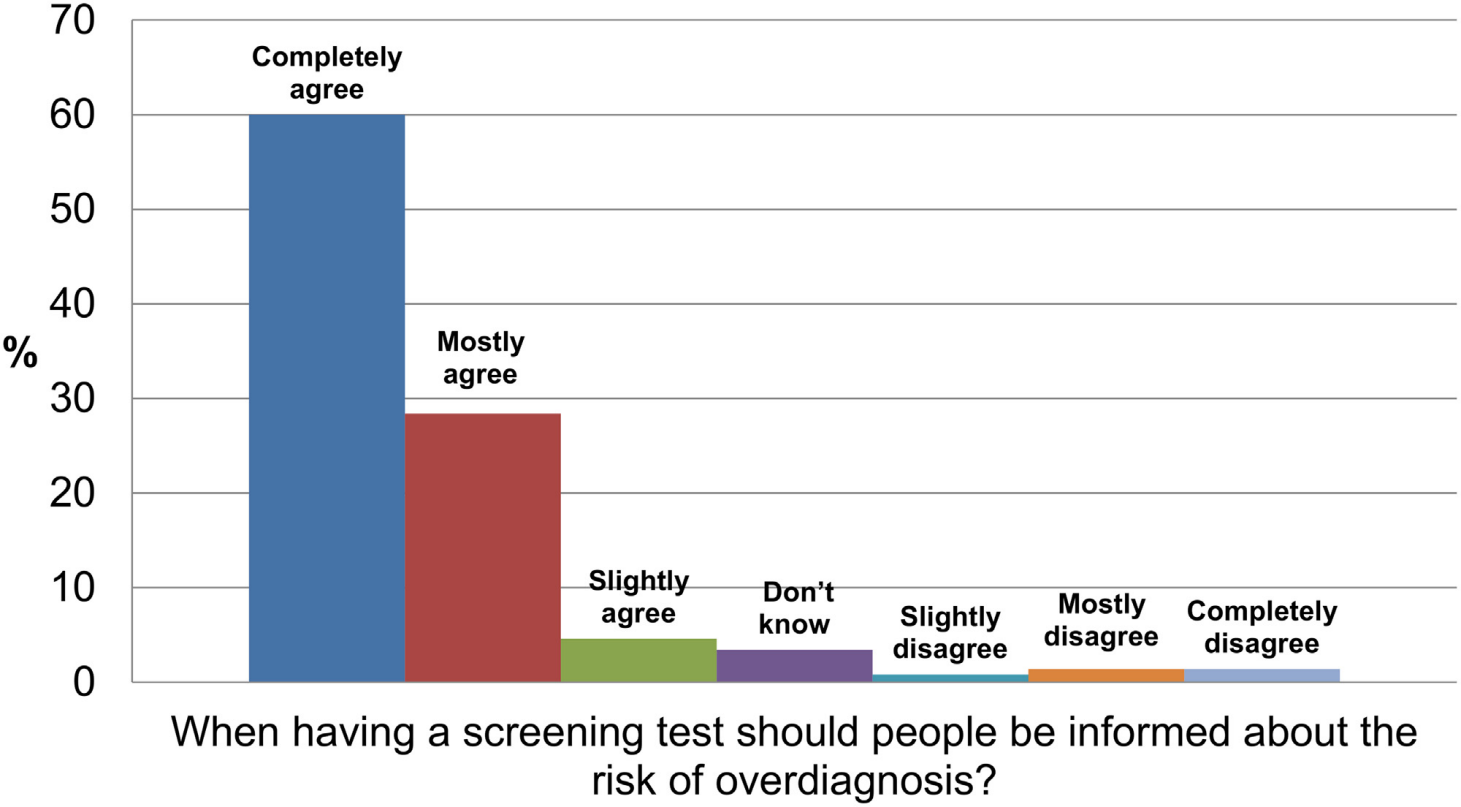
Community views about availability of information on overdiagnosis.

Of all participants, 63% (95% CI 58%-67%) said they had heard or seen the word overdiagnosis before, although following a brief explanation of the term to all participants (Table 1) the number fell to 50% (95% CI: 45%-54%). Only 10% of people said they had ever been told by a doctor that overdiagnosis was a risk of being screened or tested, (95% CI 8%-13%). Only 18% of men who reported having had prostate cancer screening (95% CI 11%-28%) and 10% of women who reported having had a mammogram (95% CI 6%-15%) said they were told about the risk of overdiagnosis. 76% of participants (95% CI 72%-80%) agreed screening tests were almost always a good idea and 93% (95% CI 90%-95%) agreed (88% completely or mostly agreed) that along with the benefits of screening, people should be informed about the risk of overdiagnosis. (Fig 2).

Asked about enthusiasm for a genetic screening test, the community was split almost equally, with 49% likely to have a screening test, (95% CI 44%-53%) and 45% unlikely,(95% CI 41%-50%). When asked to imagine the results of genetic screening tests were often uncertain and predictions potentially wrong, enthusiasm waned dramatically, with 28% likely, (95% CI 25%-33%) and 67% unlikely, (95% CI 63%-71%) to undergo tests.

**Fig 3:**
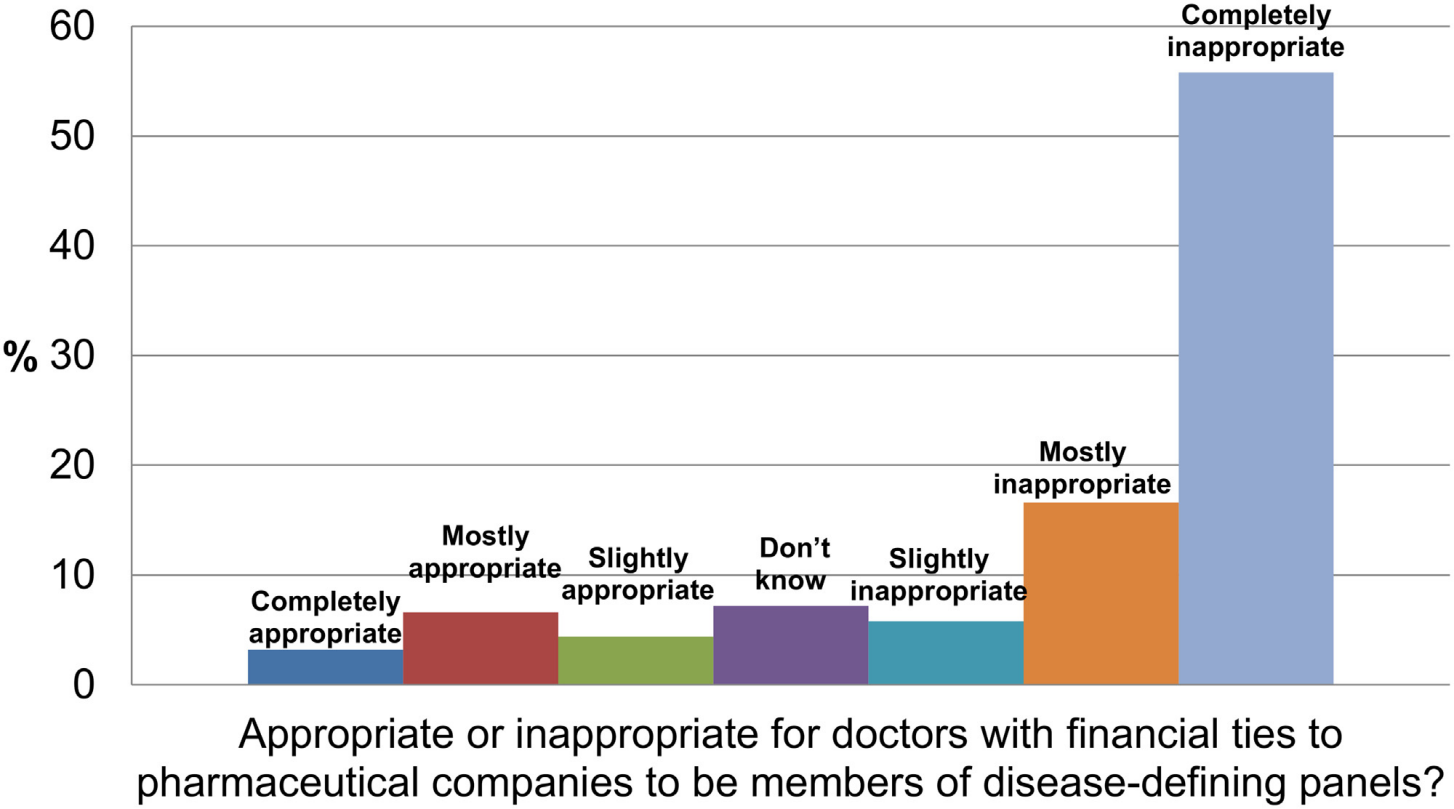
Community views about financial ties of panel members who set disease definitions.

In response to questions about panels which set disease definitions, 78% (95% CI 74%-82%) felt it inappropriate (72% completely or mostly inappropriate) for members to have financial ties to pharmaceutical companies. (Fig 3) Asked what proportion of panel members would ideally have financial ties to pharmaceutical companies, 55% (95% CI 50%-59%) said there should be no panel members with ties, 36% (95% CI 32%-40%) said a minority—less than 50%, and 5% (95% CI 3%-7%) said a majority—50% or more. At the conclusion of the survey, 81% of all participants volunteered to take part in a qualitative follow-up study. The number of refusals to answer questions was negligible.

For four key questions—unprompted awareness of overdiagnosis, whether participants had been informed about overdiagnosis, merits of routine information about overdiagnosis, and appropriateness of financial ties for disease panels—we looked for associations with 5 key demographics: age, gender, employment, education and cancer history. For gender and employment, we found no significant associations. Responses to unprompted awareness of the term overdiagnosis had significant associations with age, education status and cancer history. After re-coding multiple categories for age into two categories above and below the median age, and removing “don’t knows” and refusals, among those aged 53 and younger, 57% said yes they had heard or seen the term, while for those older than 53 years, 71% said yes, (chi-square 9.7, p = 0.002). For those with a history of cancer, 76% said yes they had heard or seen the term, and for those without 61% said yes (chi-square 5.5, p = 0.019). After re-coding the highest level of education into two categories, among those with education up to and including year 12(~age 17), 51% said they were aware of the term, while for those with a post-year 12 education the figure was 68%, (chi-square 12, p = 0.001). The only other significant association also involved education levels: those with higher levels were slightly more likely to report a doctor had informed them about overdiagnosis (13% vs 6%; chi square 5.0, p = 0.025).

The only key outcome that had more than one significant association with a demographic variable was the question about having heard of the term ‘overdiagnosis’. Therefore, age (above and below the median age), educational level (above and equal to or below year 12 attainment) and history of cancer diagnosis were used as predictors in multivariable logistic regression. All were significant. The adjusted odds ratio for reporting having heard of overdiagnosis was 1.8 for the older age group (p = 0.003, 95% CI 1.2 to 2.7), 2.2 for educational level above year 12 (p = 0.001, 95% CI 1.5 to 3.4) and 1.9 for those with a history of cancer diagnosis (p = 0.048, 95% CI 1.0 to 3.4).

## Discussion

Our community survey found a large majority of adults reporting they had not been informed about the risk of overdiagnosis attached to screening tests, and a large majority expressing the view that along with screening benefits, people should be informed of the risk of overdiagnosis. Despite strong evidence overdiagnosis is a significant risk of prostate cancer screening [6], 81% of men who reported being screened said they had not been told; among women who reported having breast cancer screening, where overdiagnosis is also now an established risk [5], 87% said they hadn’t been informed.

Over two-thirds of Australians surveyed felt it was completely or mostly inappropriate for doctors with financial ties to pharmaceutical companies to serve on panels which set disease definitions. Moreover, when asked how these panels should ideally be constituted, just over half of all participants said they should have no doctors with financial ties.

Against a background of concern that widespread promotion of genetic screening could produce new vectors for overdiagnosis [22], we found public enthusiasm might be modified if people were informed the results of screening tests could be uncertain or potentially wrong. Given the propensity of media coverage to over-promote benefits and minimise harms [27], our findings suggest routine provision of information about the limitations and potential harms of screening tests may be desirable.

Our study has several important limitations. As part of the survey methodology we axiomatically relied solely on unverified self-reports. While previous data suggests telephone survey self-reports of screening are reliable indicators of actual behaviour [28], some of the large majority who reported not being informed about overdiagnosis may in fact have been informed. Secondly, because this is a new area of inquiry, our questionnaire has not been used before, apart from the question on enthusiasm for screening taken with minor modification from a previously published national survey [17]. While new items were rigorously piloted by the research team and social research company using a multi-stage pilot process with 40 adults, and explicit efforts were made to ensure questions were not leading, we cannot exclude the possibility some responses may be influenced by the questions. A third limitation arises from the complexity of the material, though there was a strong focus on comprehensibility and clarity in questionnaire development and interviewer training.

A final limitation arises from the AAPOR response rate of 20.4% and cooperation rate of 43.8%. While modest, these rates are common and considered satisfactory for community surveys of this type. In 2012 the highly regarded Pew Research Centre stated its standard telephone surveys were achieving an AAPOR response rate of 9%, and cooperation rate of 14%, and that the 9% response rate was similar to that achieved by other major survey organisations [29]. With the outcome rates achieved there is a possibility of systematically different responses between respondents and non-respondents, though this possibility is lessened by the general representativeness of sample respondents.

Alongside limitations, the study has important strengths. In the context of growing evidence about overdiagnosis this is to our knowledge the first national telephone survey to assess how the general community reports being informed about overdiagnosis, finding both a deficit of information and a desire for it. Secondly, we gathered rare and novel data on community attitudes about the timely question of who should most appropriately be setting diagnostic criteria which determine the nature and extent of human pathology. And finally, our random sample was generally representative of the Australian community, achieved in part as a result of our dual frame method, reaching both landline-users and the fast growing demographic of mobile-only users, now estimated to be more than 20% of phone users in Australia [30], and 38% in the United States [31]. Moreover, generally negligible differences between adjusted and unadjusted results strengthen representativeness and generalizability, and potential applicability to other nations with similar demographics.

There is extremely limited data on public awareness about overdiagnosis. A small on-line survey limited to individuals who had been invited to undergo cancer screening—reported briefly as a research letter in 2013—found only 9.5% reported they’d been informed by a physician about the risk of overdiagnosis and overtreatment, and 80% felt people should be routinely informed of such screening harms [19]. Similarly our survey found only a small proportion reported being told of these harms, and 88% completely or mostly agreed people should be informed about the risk of overdiagnosis, echoing findings from a 2002 survey of around 650 Australian women, which found over 90% wanted to receive information about false results or mammogram side effects [32]. This strong community desire for information about harms is set against a backdrop of widespread enthusiasm for screening. In 2004 Schwartz and colleagues found 87% agreed routine cancer screening was almost always a good idea [17], while 76% agreed with a similar proposition in our survey a decade later.

There are mixed findings on public attitudes to financial ties between health professionals and industry. Some studies suggest trial participants want information about investigator financial ties, but are not deterred by them [20], while other studies find concern strongest where the tie brings direct benefits, such as the professional being paid research recruitment fees [21]. To our knowledge, no previous study has investigated public opinions about the pharmaceutical company ties of panels which set disease definitions. Our finding of strong public antipathy to these ties is significant and timely: in tune with Institute of Medicine recommendations to minimise and eliminate them [16] but in contrast to current reality, where many panel members have such ties [15].

In light of the limitations of this telephone survey, and the complexity of the material covered, caution in interpretation is appropriate. In 2014 researchers in the United Kingdom reported that even written information about overdiagnosis and mammography was not well understood [33]. However, despite that complexity, at the completion of our survey, around 400 of the 500 participants, ultimately shared personal details and agreed to take part in a follow-up qualitative research project, underscoring not only a positive survey experience, but suggesting a public hunger to learn more about overdiagnosis and related issues.

Responding to overdiagnosis also poses complex challenges. Some degree of overdiagnosis is inevitable whenever healthy individuals are screened [8], on-going monitoring of screening programmes may reveal new technologies that can reduce that risk [7], and early detection can offer genuine opportunities for lifestyle changes or other preventive strategies. While increasing numbers of research projects are underway worldwide investigating the nature and extent of overdiagnosis, these survey findings, notwithstanding limitations and complexities, point to the need to find ways to better communicate with the community about the problem. Not least to facilitate more informed decision making, but more broadly, as Clarke suggests [10], to enable “greater democratic participation” in shaping the future of relationships between people and their health care.

## Supporting Information

S1 File Over-diagnosis Questionnaire. (DOCX)
