# Supplementary material for: Public Opinions about Overdiagnosis: A National Community Survey
Source: PLoS One. 2015 May 20;10(5):e0125165. doi: 10.1371/journal.pone.0125165 (PMC4439083; doi:10.1371/journal.pone.0125165)
Supplement: S1 File — (DOCX) [file pone.0125165.s001.docx]

**Bond University**

**“Medical Over-diagnosis”**

16 January, 2014

Questionnaire version 8 (including pilot changes)

**[PROGRAMMER NOTE:**

- **Use standard SMS list**
- **Use standard RR1 list]**

# *INTRODUCTION & SCREENING

*(PHONE ANSWERER)

INTRO1: Good (morning/afternoon/evening) my name is (…) and I am calling from the Social Research Centre on behalf of Bond and Sydney Universities.

We’re doing a short survey across Australia about community views on the way doctors diagnose diseases. This is an emerging issue and the results will help researchers find better ways for doctors to communicate about the risks and benefits of medical tests and treatments.

1. Continue

*(LANDLINE)

INTRO2a: We would like to speak to the person in your household who is aged 18 years or over and who had the LAST birthday? Would that be you?

[REINTRODUCE IF NECESSARY: Good (morning/afternoon/evening) my name is (…) and I am calling from the Social Research Centre on behalf of Bond and Sydney Universities. We’re doing a short survey across Australia about community views on the way doctors diagnose diseases. This is an emerging issue and the results will help researchers find better ways for doctors to communicate about the risks and benefits of medical tests and treatments.]

1. Yes (continue)
2. Not a good time: appointment (MAKE APPOINTMENT)
3. Household refusal (GO TO RR1)
4. Language difficulty (NO follow up) (GO TO TERM2)
5. Wants more information before participating (GO TO INFO1)
6. No one aged 18+ in household (GO TO TERM1)
7. Queried about how telephone number was obtained (GO TO TEL_LAND)

*(MOBILE)

INTRO2b. Can I ask whether you are aged 18 years or over?

1. Yes (continue)
2. Not a good time: appointment (GO TO MOB1)
3. Mobile answerer refusal (GO TO RR1)
4. Language difficulty (NO follow up) (GO TO TERM2)
5. Wants more information before participating (GO TO INFO1)
6. Under 18 years of age (GO TO TERM1)
7. Queried about how telephone number was obtained (GO TO TEL_MOB)
8. Queried why mobile was called (GO TO TINFO_MOB)

*(IF INTRO2a=5 OR INTRO2b=5 – MORE INFO REQUIRED)

INFO1. You can find more information online from the Social Research Centre website ([www.srcentre.com.au](http://www.srcentre.com.au), on the ‘participants’ tab under ‘current surveys’. This includes links to the Bond University and the University of Sydney websites.)

1. Make appointment [PROGRAMMER NOTE: IF MOBILE GO TO MOB1, ELSE MAKE APPOINTMENT]
2. (Wants info but NO INTERNET) (COLLECT MAILING DETAILS THEN IF MOBILE GO TO MOB1, ELSE MAKE APPOINTMENT; SET UP GETDET!]

*(QUERIED HOW LANDLINE NUMBER WAS OBTAINED)

TEL_LAND Your telephone number has been chosen at random from all possible telephone numbers. We find that this is the best way to obtain a representative sample of people across Australia.

1. Snap back to previous question

*(QUERIED HOW MOBILE NUMBER WAS OBTAINED)

TEL_MOB Your mobile number was randomly generated by a computer. We’re calling mobile phones as well as landlines so we can get a representative sample of people across Australia.

1. Snap back to previous question

*(QUERIED WHY MOBILE WAS CALLED)

TINFO_MOB One of the issues currently facing telephone survey researchers in Australia is the increasing proportion of households without a landline telephone. We are calling mobile phones as well as landlines so we can get a representative sample of people across Australia.

1. Snap back to previous question

*(MOBILE SAMPLE)

MOB2. May I just check whether or not it is safe for you to take this call at the moment? If not, I am happy to call you back when it is more convenient for you.

1. Safe to take call
2. Not safe to take call
3. Respondent refusal (GO TO RR1)

*(MOBILE SAMPLE)

MOB1. Just so I know your time zone, can you tell me which state you‘re in?

1. NSW
2. VIC
3. QLD
4. SA
5. WA
6. TAS
7. NT
8. ACT
9. (Refused)

*PROGRAMMER NOTE – WRITE STATE / TERRITORY TO SAMPLE RECORD

[PREMOBAPPT IF INTRO2b=2 OR 5, OR MOB2=2 CONTINUE (mob appointment), ELSE GO TO INTRO3a]

*(MOBILE SAMPLE NEEDING APPOINTMENT)

MOB_APPT Do you want me to call you back on this number or would you prefer I call back on another phone?

1. This number (TYPE STOP, MAKE APPOINTMENT)
2. Another number (TYPE STOP, MAKE APPOINTMENT, RECORD ALT NUMBER)
3. Respondent refusal (GO TO RR1)

*(ALL)

INTRO3a: Thank you. Now, I’m just going to run some details past you about the study….

1. Continue
2. Not a good time (MAKE APPOINTMENT)
3. Respondent refusal (GO TO RR1)
4. Language difficulty (NO follow up) (GO TO TERM2)
5. More info “go to [www.srcentre.com.au](http://www.srcentre.com.au), on the ‘participants’ tab under ‘current surveys’. This includes links to the Bond University and the University of Sydney websites.” (MAKE APPOINTMENT)
6. (Wants info but NO INTERNET: collect details to mail info sheet) (MAKE APPOINTMENT)

*(ALL)

INTRO3b:

The survey has been approved by the Bond University Ethics Committees and participation is completely voluntary. You can choose not to answer any question or to cease the survey at any time. Your phone number was selected randomly, your answers will remain completely anonymous and we will not be recording this phone call. It will only take around 15 minutes to complete.

Would you like more information or would you be willing to participate in this important study?

[INTERVIEWER NOTE: If more information requested, read the abridged Participant Information Sheet. If potential participant doesn’t want to be read information, offer to provide website details and arrange an appointment.]

1. Continue
2. Not a good time (MAKE APPOINTMENT)
3. Respondent refusal (GO TO RR1)
4. More info “go to [www.srcentre.com.au](http://www.srcentre.com.au), on the ‘participants’ tab under ‘current surveys’. This includes links to the Bond University and the University of Sydney websites.” (MAKE APPOINTMENT)
5. (NO INTERNET ACCESS: arrange to send information sheet) (MAKE APPOINTMENT)

*(ALL)

MON: This call may be monitored for training and quality purposes but will NOT be recorded. Is that OK?

1. Monitor
2. Do not monitor

*(ALL)

S1a. Thank you. Now, before we begin the survey, would you mind telling me your age?

1. (Record number in years) [RANGE 18 to 105]
2. (Refused) (GO TO S1b)

*(IF S1a=2 – Refused age)

S1b. Could you please tell me which of the following age groups you are in?

1. 18-29
2. 30-49
3. 50-69
4. 70 and over
5. (Refused)

*(ALL)

S2. As we are talking about the way doctors diagnose diseases and medical treatments which affect men and women differently would you mind confirming your gender?

[IF NECESSARY: we need to ask this question of everyone to make sure we ask people the right questions throughout the survey]

[INTERVIEWER NOTE: If ‘refuse’ or ‘other’, allocate at your discretion]

1. Male
2. Female

*(ALL)

S3. What is the post code of the area in which you live?

1. (Record post code ____) [RANGE: 800 to 9999]
2. (Don’t know)(Specify suburb)
3. (Refused)

[PROGRAMMER NOTE: DERIVE ‘STATE’ WHERE RESPONDENT CAN PROVIDE POSTCODE; IF NO ELIGIBLE POSTCODE PROVIDED ALLOCATE TO ‘OTHER’ STATE]

# *SECTION A: GENERAL KNOWLEDGE ABOUT OVER-DIAGNOSIS

Just to start our survey…

*(ALL)

A1. Have you seen or heard the term ‘over-diagnosis’ before today?

[IF NECESSARY: We are discussing over-diagnosis of medical illnesses, diseases and conditions].

1. Yes
2. No
3. (Don’t know)
4. (Refused)

[PRE A2 IF A1=1 use A2i ELSE use A2ii]

*(ALL)

A2i. What do you understand the term ‘over-diagnosis’ to mean?

A2ii. What do you think the term ‘over-diagnosis’ means?

[IF NECESSARY: We are discussing over-diagnosis of medical illnesses, diseases and conditions].

1. Response Given (Specify) *(PROGRAMMER NOTE: Set up as full verbatim)

2. (Don’t know / can’t say)

3. (Refused)

*(ALL)

A3. A generally accepted view is that over-diagnosis happens when people are diagnosed with a disease that would never have harmed them. This could be due to the condition being so slow developing or them displaying only very minor symptoms.

Given this explanation, have you seen or heard the term or concept of ‘over-diagnosis’?

[IF NECESSARY: For example sometimes people are diagnosed with cancer, but that cancer would be so slow growing it would not cause them any harm in their lifetime, or a person with very mild problems may be diagnosed with a mental disorder such as ADHD.

We are discussing over-diagnosis of medical illnesses, diseases and conditions].

1. Yes
2. No
3. (Don’t know)
4. (Refused)

*(ALL)

A4. Has a doctor ever told you that healthy people can be over-diagnosed as a result of being screened or tested for a disease?

1. Yes
2. No
3. (Don’t know)
4. (Refused)

*(ALL)

A5. Routine screening means testing healthy people to find signs of diseases such as cancer.

Do you think routine screening tests for healthy people are almost always a good idea?

1. Yes
2. No
3. (Don’t know)
4. (Refused)

*(ALL)

A6. Do you agree or disagree that routine screening tests for healthy people are important for their health?

PROBE: Is that completely, mostly or SLIGHTLY agree / disagree?

(RESPONSE FRAME) (DO NOT READ OUT)

1. Completely agree
2. Mostly agree
3. Slightly agree
4. (Neither agree nor disagree)
5. Slightly disagree
6. Mostly disagree
7. Completely disagree
8. (Don’t know / Can’t say)
9. (REFUSED)

*(ALL)

A7. When healthy people are considering having a screening test - along with being told about the potential benefits of the screening test – do you agree or disagree that they should be informed about the potential risk of over-diagnosis?

[IF NECESSARY: For example a screening test for prostate or breast cancer, chronic kidney disease or other diseases and illnesses].

PROBE: Is that completely, mostly or SLIGHTLY agree / disagree?

(RESPONSE FRAME) (DO NOT READ OUT)

1. Completely agree
2. Mostly agree
3. Slightly agree
4. (Neither agree nor disagree)
5. Slightly disagree
6. Mostly disagree
7. Completely disagree
8. (Don’t know / Can’t say)
9. (REFUSED)

*(ALL)

TS1_Section A

# *SECTION B: SCREENING EXPERIENCES

*(ALL)

B0. I will now ask you a few brief questions about your experiences with medical screening tests.

[PREB1a: IF S2=2 (female) GO TO PREB2; ELSE CONTINUE]

*(S2=1 – Males only)

B1a Have you ever had a screening test, sometimes called a PSA test before for prostate cancer?

1. Yes
2. No (GO TO B3)
3. (Don’t know) (GO TO B3)
4. (Refused) (GO TO B3)

*(B1a=1 – had screening test)

B1b. Were you told about the risk of over-diagnosis (of this test)?

[IF NECESSARY: In other words, were you told by a doctor, GP, or the person who offered or administered the test that you may be diagnosed and treated for a cancer that would never have caused you any harm in your lifetime?]

1. Yes I was told
2. No I wasn’t told
3. (Don’t know)
4. (Refused)

[PREB2: IF S2=1 (male) GO TO B3; ELSE CONTINUE]

*(S2=1 – Females only)

B2a Have you ever had a mammogram to screen for breast cancer?

[IF NECESSARY: “We are interested in finding out if women have had a screening test when they didn’t have any problem in their breast, such as a lump, as opposed to having a ‘diagnostic’ mammogram, where there was a problem being investigated.]

1. Yes
2. No (GO TO B3)
3. (Don’t know) (GO TO B3)
4. (Refused) (GO TO B3)

*(B2a=1 – had screening test)

B2b. Were you told about the risk of over-diagnosis (of this test)?

[IF NECESSARY: In other words, were you told by a doctor, GP, or the person who offered or administered the test that you may be diagnosed and treated for a cancer that would never have caused you any harm in your lifetime?]

1. Yes I was told
2. No I wasn’t told
3. (Don’t know)
4. (Refused)

*(ALL)

B3. Have you ever had a genetic screening test?

[IF NECESSARY: A genetic screening test, or DNA testing, seeks to identify inherited diseases or diseases passed on through blood relations]

1. Yes
2. No
3. (Don’t know)
4. (Refused)

*(ALL)

TS2_Section B

# *SECTION C: INTEREST IN GENETIC SCREENING

*(ALL)

C1. Imagine that there was a genetic screening test which could analyse your genes and identify all the diseases you may ever get, for which some had effective treatments and some did not. Would you be likely or unlikely to have that screening test?

PROBE: Is that COMPLETELy, mostly or SOMEWHAT LIKELY / UNLIKELY?

(RESPONSE FRAME)

1. Completely likely
2. Mostly likely
3. Somewhat likely
4. (Neither likely nor unlikely)
5. Somewhat unlikely
6. Mostly unlikely
7. Completely unlikely
8. (Don’t know)
9. (Refused)

*(ALL)

C2. Imagine now that the results of the genetic screening test were often uncertain, and the predictions could be wrong. Would you be likely or unlikely then to have that screening test?

PROBE: Is that COMPLETELy, mostly or SOMEWHAT LIKELY / UNLIKELY?

(RESPONSE FRAME)

1. Completely likely
2. Mostly likely
3. Somewhat likely
4. (Neither likely nor unlikely)
5. Somewhat unlikely
6. Mostly unlikely
7. Completely unlikely
8. (Don’t know)
9. (Refused)

*(ALL)

TS3_Section C

# *SECTION D: BREAST SCREENING (MALES VS FEMALES)

*(ALL)

D0. In this next section I’m going to describe a particular scenario about screening for cancer, then ask you some questions about that scenario. Sometimes people can find these questions personal or sensitive. If you are unsure how to answer or do not want to answer any question please let me know and I will move on.

[PROGRAMMER NOTE: create dummy variable to randomly assign males and females to relevant Option 1 and Option 2. Each respondent to respond to one scenario.]

*(ALL) [PROGRAMMER NOTE: All respondents need to be assigned a code at RAND; minimum of 100 respondents per code]

RAND: “Random assignment of males and females to Option 1 and Option 2”

1. S2=2 + random assignment (Female, Option 1)
2. S2=2 + random assignment (Female, Option 2)
3. S2=1 + random assignment (Male, Option 3)
4. S2=1 + random assignment (Male, Option 4)

[PRED1: IF RAND=1, CONTINUE. ELSE GO TO PRED5]

*(RAND=1 – Female, Option 1)

D1. Breast screening (mammograms) detects abnormal changes of cells in the breast as well as finding breast cancers. In some women these abnormal cells can progress to invasive cancer and in others they do not. It’s estimated that if left untreated about one-third may progress to breast cancer over 10 years or more. That means that for about two-thirds of women these abnormal cells may not become cancer.

Imagine you had an abnormal breast screen and follow-up tests showed that there were abnormal cells found in your breast.

How concerned would you be about your result? Would you say…

[IF NECESSARY: ‘Invasive’ cancer means potentially life threatening.]

(RESPONSE FRAME) (READ OUT)

1. Extremely concerned
2. Moderately concerned
3. (Neither concerned nor unconcerned)
4. Not really concerned
5. Not concerned at all
6. (Don’t know)
7. (Refused)
8. (Respondent does not understand terminology / issues) (GO TO TS4)

*(RAND=1 – Female, Option 1)

D2. Abnormal breast cells are usually treated by surgery, radiation or drugs as in the case of breast cancer. Another approach is called watchful waiting, where doctors closely monitor the abnormal breast cells with regular mammograms and only treat if cells become more abnormal.

If research shows that watchful waiting is a safe and effective option, how do you think you would prefer to manage these abnormal cells? Would you say…

[IF NECESSARY: If people want more information on exactly what watchful waiting would entail - say, that’s what research would determine.]

(RESPONSE FRAME) (READ OUT)

1. Definitely prefer treatment
2. Probably prefer treatment
3. (Prefer to do nothing)
4. Probably prefer watchful waiting (close monitoring by doctors)
5. Definitely prefer watchful waiting (close monitoring by doctors)
6. (Don’t know)
7. (Refused)
8. (Respondent does not understand terminology / issues) (GO TO TS4)

*(RAND=1 – Female, Option 1)

D3. Thinking again about the previous scenario, if these abnormal cells in your breast were instead called pre-invasive breast cancer cells (rather than abnormal cells), would you be more concerned or less concerned about your screening test result?

(RESPONSE FRAME)

1. More concerned
2. (No difference)
3. Less concerned
4. (Don’t know)
5. (Refused)
6. (Respondent does not understand terminology / issues) (GO TO TS4)

*(RAND=1 – Female, Option 1)

D4. And if research shows that watchful waiting is a safe and effective option, how do you think you would prefer to manage these pre-invasive breast cancer cells? Would you say…

(RESPONSE FRAME) (READ OUT)

1. Definitely prefer treatment
2. Probably prefer treatment
3. (Prefer to do nothing)
4. Probably prefer watchful waiting (close monitoring by doctors)
5. Definitely prefer watchful waiting (close monitoring by doctors)
6. (Don’t know)
7. (Refused)
8. (Respondent does not understand terminology / issues) (GO TO TS4)

[PRED5: IF RAND=2, CONTINUE. ELSE GO TO PRED9]

*(RAND=2 – Female, Option 2)

D5. Breast screening (mammograms) detects pre-invasive breast cancer cells in the breast as well as finding breast cancers. In some women these pre-invasive breast cancer cells can progress to invasive cancer and in others they do not. It’s estimated that if left untreated about one-third may progress to breast cancer over 10 years or more. That means that for about two-thirds of women these pre-invasive breast cancer cells may not become cancer.

Imagine you had a breast screen and follow-up tests showed that there were pre-invasive breast cancer cells found in your breast.

How concerned would you be about your result? Would you say…

[IF NECESSARY: ‘Invasive’ cancer means potentially life threatening.]

(RESPONSE FRAME) (READ OUT)

1. Extremely concerned
2. Moderately concerned
3. (Neither concerned nor unconcerned)
4. Not really concerned
5. Not concerned at all
6. (Don’t know)
7. (Refused)
8. (Respondent does not understand terminology / issues) (GO TO TS4)

*(RAND=2 – Female, Option 2)

D6. Pre-invasive breast cancer cells are usually treated by surgery, radiation or drugs as in the case of breast cancer. Another approach is called watchful waiting, where doctors closely monitor the pre-invasive breast cancer cells with regular mammograms and only treat if cells become more invasive.

If research shows that watchful waiting is a safe and effective option, how do you think you would prefer to manage these pre-invasive breast cancer cells? Would you say…

[IF NECESSARY: If people want more information on exactly what watchful waiting would entail - say, that’s what research would determine.]

(RESPONSE FRAME) (READ OUT)

1. Definitely prefer treatment
2. Probably prefer treatment
3. (Prefer to do nothing)
4. Probably prefer watchful waiting (close monitoring by doctors)
5. Definitely prefer watchful waiting (close monitoring by doctors)
6. (Don’t know)
7. (Refused)
8. (Respondent does not understand terminology / issues) (GO TO TS4)

*(RAND=2 – Female, Option 2)

D7. Thinking again about the previous scenario, if these pre-invasive breast cancer cells were instead called abnormal cells (rather than pre-invasive cells), would you be more concerned or less concerned about your screening test result?

(RESPONSE FRAME)

1. More concerned
2. (No difference)
3. Less concerned
4. (Don’t know)
5. (Refused)
6. (Respondent does not understand terminology / issues) (GO TO TS4)

*(RAND=2 – Female, Option 2)

D8. And if research shows that watchful waiting is a safe and effective option, how do you think you would prefer to manage these abnormal cells? Would you say…

(RESPONSE FRAME) (READ OUT)

1. Definitely prefer treatment
2. Probably prefer treatment
3. (Prefer to do nothing)
4. Probably prefer watchful waiting (close monitoring by doctors)
5. Definitely prefer watchful waiting (close monitoring by doctors)
6. (Don’t know)
7. (Refused)
8. (Respondent does not understand terminology / issues) (GO TO TS4)

[PRED9: IF RAND=3, CONTINUE. ELSE GO TO PRED13]

*(RAND=3 – Male, Option 3)

D9. Breast screening (mammograms) detects abnormal changes of the cells in the breast as well as finding breast cancers. In some women these abnormal cells can progress to invasive cancer and in others they do not. It’s estimated that if left untreated about one-third may progress to breast cancer over 10 years or more. That means that for about two-thirds of women these abnormal cells may not become cancer.

Imagine your wife, daughter, mother or close female friend had an abnormal breast screen and follow-up tests showed that there were abnormal cells found in her breast.

How concerned would you be about her result? Would you say…

[IF NECESSARY: ‘Invasive’ cancer means potentially life threatening.]

(RESPONSE FRAME) (READ OUT)

1. Extremely concerned
2. Moderately concerned
3. (Neither concerned nor unconcerned)
4. Not really concerned
5. Not concerned at all
6. (Don’t know)
7. (Refused)
8. (Respondent does not understand terminology / issues) (GO TO TS4)

*(RAND=3 – Male, Option 3)

D10. Abnormal breast cells are usually treated by surgery, radiation or drugs as in the case of breast cancer. Another approach is called watchful waiting, where doctors closely monitor the abnormal breast cells with regular mammograms and only treat if cells become more abnormal.

If research shows that watchful waiting is a safe and effective option, how do you think you would prefer she manage these abnormal cells? Would you say…

[IF NECESSARY: If people want more information on exactly what watchful waiting would entail - say, that’s what research would determine.]

(RESPONSE FRAME) (READ OUT)

1. Definitely prefer treatment
2. Probably prefer treatment
3. (Prefer to do nothing)
4. Probably prefer watchful waiting (close monitoring by doctors)
5. Definitely prefer watchful waiting (close monitoring by doctors)
6. (Don’t know)
7. (Refused)
8. (Respondent does not understand terminology / issues) (GO TO TS4)

*(RAND=3 – Male, Option 3)

D11. Thinking again about the previous scenario and the same person, if these abnormal breast cells were now called pre-invasive breast cancer cells (rather than abnormal cells), would you be more concerned or less concerned about her screening test result?

(RESPONSE FRAME)

1. More concerned
2. (No difference)
3. Less concerned
4. (Don’t know)
5. (Refused)
6. (Respondent does not understand terminology / issues) (GO TO TS4)

*(RAND=3 – Male, Option 3)

D12. And if research shows that watchful waiting is a safe and effective option, how do you think you would prefer your wife, daughter, mother or close female friend manage these pre-invasive breast cancer cells? Would you say…

(RESPONSE FRAME) (READ OUT)

1. Definitely prefer treatment
2. Probably prefer treatment
3. (Prefer to do nothing)
4. Probably prefer watchful waiting (close monitoring by doctors)
5. Definitely prefer watchful waiting (close monitoring by doctors)
6. (Don’t know)
7. (Refused)
8. (Respondent does not understand terminology / issues) (GO TO TS4)

[PRED13: IF RAND=4, CONTINUE. ELSE GO TO TS4_SECTION D]

*(RAND=4 – Male, Option 4)

D13. Breast screening (mammograms) detects pre-invasive breast cancer cells in the breast as well as finding breast cancers. In some women these pre-invasive breast cancer cells can progress to invasive cancer and in others they do not. It’s estimated that if left untreated about one-third may progress to breast cancer over 10 years or more. That means that for about two-thirds of women these pre-invasive breast cancer cells may not become cancer

Imagine your wife, daughter, mother or close female friend had a breast screen and follow-up tests showed that there were pre-invasive breast cancer cells found in her breast.

How concerned would you be about her result? Would you say…

[IF NECESSARY: ‘Invasive’ cancer means potentially life threatening.]

(RESPONSE FRAME) (READ OUT)

1. Extremely concerned
2. Moderately concerned
3. (Neither concerned nor unconcerned)
4. Not really concerned
5. Not concerned at all
6. (Don’t know)
7. (Refused)
8. (Respondent does not understand terminology / issues) (GO TO TS4)

*(RAND=4 – Male, Option 4)

D14. Pre-invasive breast cancer cells are usually treated by surgery, radiation or drugs as in the case of breast cancer. Another approach is called watchful waiting, where doctors closely monitor the pre-invasive breast cancer cells with regular mammograms and only treat if cells become more invasive.

If research shows that watchful waiting is a safe and effective option, how do you think you would prefer she manage these pre-invasive breast cancer cells? Would you say…

[IF NECESSARY: If people want more information on exactly what watchful waiting would entail - say, that’s what research would determine.]

(RESPONSE FRAME) (READ OUT)

1. Definitely prefer treatment
2. Probably prefer treatment
3. (Prefer to do nothing)
4. Probably prefer watchful waiting (close monitoring by doctors)
5. Definitely prefer watchful waiting (close monitoring by doctors)
6. (Don’t know)
7. (Refused)
8. (Respondent does not understand terminology / issues) (GO TO TS4)

*(RAND=4 – Male, Option 4)

D15. Thinking again about the previous question and the same person, if these pre-invasive breast cancer cells were now called abnormal cells (rather than pre-invasive cells), would you be more concerned or less concerned about her screening test result?

(RESPONSE FRAME)

1. More concerned
2. (No difference)
3. Less concerned
4. (Don’t know)
5. (Refused)
6. (Respondent does not understand terminology / issues) (GO TO TS4)

*(RAND=4 – Male, Option 4)

D16. And if research shows that watchful waiting is a safe and effective option, how do you think you would prefer your wife, daughter, mother or close female friend manage these abnormal cells? Would you say…

(RESPONSE FRAME) (READ OUT)

1. Definitely prefer treatment
2. Probably prefer treatment
3. (Prefer to do nothing)
4. Probably prefer watchful waiting (close monitoring by doctors)
5. Definitely prefer watchful waiting (close monitoring by doctors)
6. (Don’t know)
7. (Refused)
8. (Respondent does not understand terminology / issues) (GO TO TS4)

*(ALL)

TS4_Section D

# *SECTION E: DISEASE DEFINITION

*(ALL)

E0. Next, I’d like to ask you a couple of questions about the way diseases are defined.

1. Continue

*(ALL)

E1. From time to time, doctors who specialise in a particular disease will come together to discuss the characteristics of that disease, to decide who should be diagnosed with it and who requires treatment for it. These are called panels and currently some doctors on these panels HAVE financial ties with pharmaceutical companies who market drugs for that disease and some DO NOT.

Is it appropriate or inappropriate for doctors who HAVE financial ties with pharmaceutical companies to be members of these panels?

PROBE: Is that COMPLETELy, mostly or slightly APPROPRIATE / INAPPROPRIATE?

(IF NECESSARY: Financial ties mean do paid work such as being a speaker or a consultant)

(RESPONSE FRAME) (DO NOT READ OUT)

1. Completely appropriate
2. Mostly appropriate
3. Slightly appropriate
4. (Neither appropriate nor inappropriate)
5. Slightly inappropriate
6. Mostly inappropriate
7. Completely inappropriate
8. (Don’t know)
9. (Refused)

*(ALL)

E2. Sometimes, these panels decide to change the definition of a disease in a way that means larger or smaller numbers of people may be treated for it. A recent study found on average, roughly three-quarters of doctors on these panels had financial ties with the pharmaceutical companies selling medicines for the same diseases.

Based on this knowledge, how appropriate or inappropriate is it for doctors with financial ties to pharmaceutical companies who market drugs for that disease to be on these panels?

PROBE: Is that COMPLETELy, mostly or slightly APPROPRIATE / INAPPROPRIATE?

(RESPONSE FRAME)

1. Completely appropriate
2. Mostly appropriate
3. Slightly appropriate
4. (Neither appropriate nor inappropriate)
5. Slightly inappropriate
6. Mostly inappropriate
7. Completely inappropriate
8. (Don’t know)
9. (Refused)

*(ALL)

E3. Ideally, what proportion of the panel should be made up of doctors with financial ties to pharmaceutical companies who market drugs for that disease?

(READ OUT)

1. None (0%)
2. A minority - less than 50%
3. A majority - 50% or more
4. (Don’t care)
5. (Don’t know)
6. (Refused)

*(ALL)

TS5_Section E

# *SECTION F: EXPERIENCE WITH CANCER

*(ALL)

F0. We’re almost finished. Now I’m going to ask you a few brief questions about your experiences with cancer and cancer screening. Sometimes people can find these questions quite personal or sensitive. If you prefer not to answer any question, please let me know and I will move on.

1. Continue

[PREF1:

IF B1a=1 DISPLAY “have had a PSA test to screen for prostate cancer”

IF B1a=2 DISPLAY “have NOT had a PSA test to screen for prostate cancer”

IF B1a=3 DISPLAY “didn’t know if you had been screened for prostate cancer”

IF B1a=4 DISPLAY “would prefer not to say if you had been screened for prostate cancer”

IF B2a=1 DISPLAY “have had a mammogram to screen for breast cancer”

IF B2a=2 DISPLAY “have NOT had a mammogram to screen for breast cancer”

IF B1a=3 DISPLAY “didn’t know if you had been screened for breast cancer”

IF B1a=4 DISPLAY “would prefer not to say if you had been screened for breast cancer”]

*(ALL)

F1. Earlier you mentioned that you < have had a PSA test to screen for prostate cancer / have NOT had a PSA test to screen for prostate cancer / didn’t know if you had been screened for prostate cancer / would prefer not to say if you had been screened for prostate cancer / have had a mammogram to screen for breast cancer / have NOT had a mammogram to screen for breast cancer / didn’t know if you had been screened for breast cancer / would prefer not to say if you had been screened for breast cancer>.

Have you been screened for other forms of cancer?

1. Yes
2. No
3. (Don’t know)
4. (Refused)

*(ALL)

F2a. Have you ever been diagnosed with cancer?

1. Yes
2. No (GO TO F3a)
3. (Don’t know) (GO TO F3a)
4. (Refused) (GO TO F3a)

*(IF F2a=1 – has been diagnosed with cancer)

F2b. What type of cancer?

(ACCEPT MULTIPLES) (DO NOT READ OUT UNLESS REQUIRED)

1. Bowel
2. Breast
3. Cervical
4. Lung
5. Lymphoma
6. Melanoma
7. Prostate
8. Response Given (Specify) *(PROGRAMMER NOTE: Set up as full verbatim)
9. (Don’t know)
10. (Refused)

*(ALL)

F3a. Have any of your immediate family, that is your parents, siblings or children, ever been diagnosed with cancer?

1. Yes
2. No (GO TO TS6_Section F)
3. (Don’t know) (GO TO TS6_Section F)
4. (Refused) (GO TO TS6_Section F)

*(IF F3a=1 – has a family history of cancer)

F3b. What’s their relationship to you?

(SELECT MULTIPLE)

1. Mother
2. Father
3. Sister
4. Brother
5. Daughter
6. Son
7. (Don’t know / can’t say)
8. (Refused)

*(IF F3a=1 – has a family history of cancer)

F3c. What type or types of cancer were they diagnosed with?

(ACCEPT MULTIPLES) (DO NOT READ OUT UNLESS REQUIRED)

1. Bowel
2. Breast
3. Cervical
4. Lung
5. Lymphoma
6. Melanoma
7. Prostate
8. Response Given (Specify) *(PROGRAMMER NOTE: Set up as full verbatim)
9. (Don’t know)
10. (Refused)

*(ALL)

TS6_Section F

# *SECTION G: DEMOGRAPHICS

*(ALL)

G0. Now I would like to ask you a few demographic questions to make sure we speak with a good cross-section of the community. Again, I’d like to assure you that everything you tell me today is anonymous.

*(ALL)

G1. Are you of Aboriginal and/or Torres Strait Islander origin?

1. Yes
2. No

*(ALL)

G2. What is the main language you speak at home?

1. English
2. Other (specify________)

*(ALL)

G5. What is your employment status?

1. Permanent or on-going
2. Casual/temporary (with no paid sick or annual leave)
3. Fixed-term contract
4. Self-employed
5. (On paid leave: e.g. maternity leave)
6. Unemployed (e.g. looking or not looking for work)
7. Not working / not in the labour force (e.g. student, home duties, retired)

*(ALL)

G6. Are you now or have you ever worked as a health professional? This includes Doctors, Specialists, Nurses or Pharmacists.

1. Yes
2. No

*(ALL)

G7. What is the highest level of education that you have completed?

(INTERVIEWER NOTE: Prompt if year 12 or below: Have you completed TAFE, trade or apprenticeship qualifications?)

(RESPONSE FRAME])

1. Postgraduate Degree
2. Graduate Diploma/Graduate Certificate
3. Bachelor Degree
4. Advanced Diploma/Diploma
5. Certificate III/IV
6. Certificate I/II
7. Certificate not further defined
8. Year 12
9. Year 11
10. Year 10 or below
11. Level not determined
12. (Don’t know)
13. (Refused)

*(ALL)

TS7_Section G

# *SECTION H: DUAL FRAME WEIGHTING

[PRESMP1 IF SAMPLE=LANDLINE CONTINUE, ELSE GO TO PRESMP3]

*(LANDLINE SAMPLE)

SMP1. How many residential phone numbers do you have in your household, not including lines dedicated to faxes, modems or business phone numbers? Do not include mobile phones.

(IF NECESSARY: How many individual LANDLINE numbers are there at your house that you can use to make and receive telephone calls?)

1. Number of lines given (Specify________) RECORD WHOLE NUMBER (ALLOWABLE RANGE 1 TO 99) *(DISPLAY “UNLIKELY RESPONSE” IF = >3)
2. Don’t know/ Not stated (PROGRAMMER NOTE: RECORD IN DATA AS 999)
3. Refused (PROGRAMMER NOTE: RECORD IN DATA AS 888)

*(LANDLINE SAMPLE)

SMP2 Do you also have a working mobile phone?

1. Yes (GO TO SMP5)
2. No (GO TO SMP5)
3. (Don’t know) (GO TO SMP5)
4. (Refused) (GO TO SMP5)

[PRESMP3 IF SAMPLE=MOBILE CONTINUE, ELSE GO TO SMP5]

*(MOBILE SAMPLE)

SMP3 Is there at least one working fixed line telephone inside your home that is used for making and receiving calls?

1. Yes
2. No (GO TO SMP5)
3. (Don’t know) (GO TO SMP5)
4. (Refused) (GO TO SMP5)

*(SMP3=1 - MOBILE SAMPLE, HAS AT LEAST ONE WORKING FIXED LINE IN HOUSEHOLD)

SMP4 How many residential phone numbers do you have in your household, not including lines dedicated to faxes, modems or business phone numbers. Do not include mobile phones.

(IF NECESSARY: How many individual LANDLINE numbers are there at your house that you can use to make and receive telephone calls?)

1. Number of lines given (Specify________) RECORD WHOLE NUMBER (ALLOWABLE RANGE 1 TO 99) *(DISPLAY “UNLIKELY RESPONSE” IF = >3)
2. Don’t know/ Not stated (PROGRAMMER NOTE: RECORD IN DATA AS 999)
3. Refused (PROGRAMMER NOTE: RECORD IN DATA AS 888)

*(ALL)

SMP5 And how many people in your household are aged 18 years or over?

1. One
2. Two or more (Specify) [ALLOWABLE RANGE 2-6]
3. (Don’t know)
4. (Refused)

*(ALL)

TELDUM (COMPUTE TELEPHONE STATUS)

1. Mobile only (SMP3=2,3,4)
2. Landline only (SMP2=2,3,4)
3. Dual user (SMP2=1 or SMP3=1)

# *CLOSE & RECONTACT

*(ALL)

CLOSE0: This brings us to the end of the survey questions. Just before we finish...

1. Continue

*(ALL)

REC1. The University of Sydney are planning to conduct another telephone survey, with similar questions to the ones you answered today, within the next 12 months. Would you be interested in being a potential participant in this future study?

(IF NECESSARY: Saying “yes” at this stage means you may be invited, but you will not be obliged to participate)

1. Yes (GO TO REC2)
2. No (GO TO END1a)

*(REC1=1 – agrees to recontact)

REC2. And can I confirm that you consent to the Social Research Centre passing your contact details (name and telephone phone number) and survey responses to Sydney University so that they will be able to contact you for the future study?

(IF NECESSARY: All of your information will be sent securely to the University of Sydney researchers and used for research purposes only. Survey responses would need to be passed on to the University of Sydney to enable re-contact based on views and experiences)

1. Yes (GO TO REC3name)
2. No (GO TO END1a)

*(REC2=1 – Agrees to recontact & to passing of details and responses)

REC3name Can you please tell me your name?

1. (Specify_______)

REC3telnum. Is this the phone number you’d like the researchers to contact you on? (If no: can you please tell me a preferred phone number?)

1. Yes
2. No – ENTER NEW TELNUM (INCLUDE AREA CODE)

RECaltnum As this survey will be conducted sometime over the next 12 months, do you have an alternative number you could give us (such as a mobile phone), just in case we can’t reach you on this phone number?

1. Yes – ENTER ALTERNATE NUM (INCLUDE AREA CODE)
2. No

*(ALL)

END1a. And finally, is over-diagnosis something you would like to know more about?

1. Yes (GO TO END1B)
2. No (GO TO END2)
3. (Don’t know) (GO TO END2)
4. (Refused) (GO TO END2)

*(IF End1a=1 – Yes would like to know more)

END1b. You can find more information on the not-for-profit website ‘Preventing Over-diagnosis’, at [www.preventingoverdiagnosis.net](http://www.preventingoverdiagnosis.net))

1. Continue

*(ALL)

END2. Thank you for your involvement in this survey. All of the information you provided today will be kept secure and only used for research purposes.

Just in case you missed it my name is (…) and this survey was conducted on behalf of Bond and Sydney Universities. If you have any questions there is a phone number I can give you if you like…..

1. Wants contact details (GO TO END3)
2. Does not want contact details (GO TO CLOSE1)

*(END2=1 - WANTS CONTACT DETAILS)

END3.

Questions about who is conducting the survey and how your telephone number was obtained: The Social Research Centre, Phone: 1800 023 040

Questions concerning the manner in which this research is being conducted - Bond University Human Research Ethics Committee, c/o Bond University Office of Research Services. Bond University, Gold Coast, 4229 Phone: +61 7 5595 4194 Fax: +61 7 5595 1120 Email: buhrec@bond.edu.au

If you have any queries or would like to be informed about the summary of research findings, please contact: Jenny Doust (Principal Investigator) Centre for Research in Evidence-Based Practice, Faculty of Health Sciences and Medicine, Bond University, Gold Coast, Queensland, Australia, 4229, Phone: 07 5595 5518; Email: [jdoust@bond.edu.au](mailto:jdoust@bond.edu.au)

Cancer Council National Helpline:131120

***(ALL)**

**CLOSE1. Thank you very much for your time.**

*(ALL)

TS8_CLOSE

# *TERMINATION SCRIPTS

TERM1. Thank you anyway but we need to speak with people who are aged 18 years and over.

TERM2. Thank you for your time.

**ALLTERM**

| **Code** | **Definition** | **Description** | **SUR category** |
| --- | --- | --- | --- |
| 1 |  | Completed interview | Interview |
| 2 | INTRO2a=3 | Household refusal | Refusal |
| 3 | INTRO2a=4 | Language other than English | Screen out |
| 4 | INTRO2a=6 | No one aged 18+ in household | Screen out |
| 5 | INTRO2b=3 | Mobile answerer refusal | Refusal |
| 6 | INTRO2b=4 | Language other than English | Screen out |
| 7 | INTRO2b=6 | Under 18 years | Screen out |
| 8 | MOB2=3 | Respondent refusal | Refusal |
| 9 | MOB_APPT=3 | Respondent refusal | Refusal |
| 10 | INTRO3a=3 | Respondent refusal | Refusal |
| 11 | INTRO3a=4 | Language other than English | Screen out |
| 12 | INTRO3b=3 | Respondent refusal | Refusal |
| 13 | All other | Midway termination | Refusals |
